# Reintroduced Grey Crowned Cranes (*Balearica regulorum*) Exhibit Reduced Dispersal and Smaller Home Ranges than Wild Conspecifics in Rwanda

**DOI:** 10.3390/ani16010006

**Published:** 2025-12-19

**Authors:** Deo Ruhagazi, Olivier Nsengimana, Placide Masengesho, Bernard Ndayisaba, Jean Ferus Niyomwungeri, Laura E. Peirson, Richard Muvunyi, Kirsten Szala-Krotkov, Curtice R. Griffin

**Affiliations:** 1 Rwanda Wildlife Conservation Association, Kigali P.O. Box 5427, Rwanda; deo@rwandawildlife.org (D.R.); olivier@rwandawildlife.org (O.N.); placide@rwandawildlife.org (P.M.); bernard@rwandawildlife.org (B.N.);; 2 Department of Conservation Medicine, Columbus Zoo and Aquarium, Powell, OH 43065, USA; peirson.laura@gmail.com; 3 Rwanda Development Board, Department of Conservation, Kigali P.O. Box 6239, Rwanda; richard.muvunyi@rdb.rw; 4 Department of Environmental Conservation, University of Massachusetts Amherst, Amherst, MA 01003, USA; kszalakrotko@umass.edu

**Keywords:** captive-rescued cranes, conservation translocation, GPS telemetry, habitat use, movement ecology, post-release adaptation, site fidelity, species recovery

## Abstract

Reintroductions are essential in wildlife conservation, but their success depends not only on demographic outcomes such as survival and reproduction, but also on behavioral adjustments that determine integration into the wild. The Grey Crowned Crane (*Balearica regulorum*), an endangered species, has suffered dramatic declines due to habitat loss, disturbance, and illegal capture. In Rwanda, a large-scale amnesty rescued 242 cranes from captivity, with 166 rehabilitated and released into Akagera National Park. This study used GPS-GSM telemetry to investigate post-release movement of reintroduced captive-rescued cranes in comparison with wild individuals. Twenty-one cranes (10 reintroduced, 11 wild) were tracked for 5–1118 days, with 15 individuals meeting criteria for detailed analyses. Reintroduced cranes exhibited reduced dispersal, with only 2 of 7 dispersing compared to 7 of 9 wild cranes, and typically after much longer residence at the release site. Reintroduced cranes also had smaller maximum displacements (median 8.4 km) and smaller home ranges (median 3.3 km^2^) relative to those of wild cranes (median 24.0 km and median 22.6 km^2^, respectively). These findings suggest that supplemental feeding and extended captivity (2 days to 10 years) of reintroduced cranes strongly influenced their high site fidelity and movement patterns. While limited dispersal may benefit short-term survival, long-term monitoring of survival, reproduction, and demographic trends is critical for assessing whether reintroduction achieves population recovery.

## Simple Summary

Grey Crowned Cranes are beautiful, endangered birds found across eastern and southern Africa. In Rwanda, many cranes had been captured illegally as chicks and juveniles and kept in captivity as pets for years. To save them, conservationists rescued the birds, rehabilitated them, and released them back into the wild. However, it was not known how these formerly captive cranes would behave compared to wild cranes, especially in terms of how far they travel and where they live. In this study, we tracked both rescued and wild cranes using GPS technology to follow their movements. We found that the rescued cranes stayed much closer to their release site and used smaller areas than wild cranes, which traveled longer distances and lived over wider ranges. The rescued cranes’ behavior was likely influenced by their history in captivity and by the supplemental food provided after release. These results show that rescued cranes may take longer to adjust to life in the wild but can survive well when supported in suitable habitats. This research provides valuable guidance for conservation programs aiming to return endangered birds to their natural environments and helps ensure the survival of Grey Crowned Cranes for future generations.

## 1. Introduction

The reintroduction of wildlife is a widely used conservation strategy aimed at restoring species to parts of their historical range [1,2]. While reintroduction programs commonly focus on population-level metrics such as survival, reproduction and growth [3], individual behavioral responses—particularly movement—are also critical for evaluating success. Movement ecology provides valuable insights into how animals adjust to novel environments, influencing site fidelity, survival and long-term integration. It also reveals individual variation, including behavioral traits that may affect stress responses and fitness [4,5]. Among these, dispersal plays a central role, as movements away from release sites can influence both habitat access and survival [4,6]. A better understanding of these processes is essential for adaptive management of reintroduction programs [1,7].

Several crane species have been reintroduced into the wild with mixed success [8]. In the United Kingdom, Common Cranes *Grus grus* have established a self-sustaining population with high survival rates [9]. The reintroduction of Mississippi Sandhill Cranes *Grus canadensis pulla* showed increased population numbers [10], although low reproduction and survival rates remain obstacles to self-sustainability [11,12]. Releases of captive-reared subadult Greater Sandhill Cranes *Grus canadensis tabida* were used to test the feasibility of establishing a non-migrating population [13]. The Whooping Crane *Grus americana* population increased despite continued low productivity and survival [14,15,16]. However, reintroductions of Siberian Cranes *Grus leucogeranus* did not increase wild population size, and post-release survival was low [17]. In southeastern Russia, reintroduced Red-crowned *Grus japonensis* and White-naped Cranes *Grus vipio* supplemented existing wild populations [18]. All these reintroductions involved either eggs or cranes raised in captivity. To our knowledge, no published studies have examined the reintroduction of cranes that were rescued from captivity, rehabilitated, and returned to the wild.

The Grey Crowned Crane, *Balearica regulorum*, is an endangered species that was once widespread throughout eastern and southern Africa [19]. Populations have fallen sharply due to habitat destruction, human disturbance, and illegal trade [20,21]. The species is monogamous, and its breeding depends on the rains, with most nesting occurring during the wetter seasons. Grey crowned cranes do not migrate but undertake varying local and seasonal movements based on rainfall, food supply, and available nesting sites. Although several studies have examined habitat use, their movement ecology is still poorly understood and has been identified as a conservation priority [21]. In Rwanda, threats to Grey Crowned Cranes have been particularly acute, with widespread illegal capture for private ownership, with many birds kept in captivity in homes and hotels [21,22]. In 2014, the Rwanda Development Board launched a national amnesty program, resulting in the rescue of 242 cranes from captivity. Of these, 166 were quarantined, rehabilitated and reintroduced into Akagera National Park by the Rwanda Wildlife Conservation Association (RWCA), in collaboration with Akagera Management Company and the Rwanda Development Board [23].

Existing research on Grey Crowned Crane movements has primarily addressed seasonal shifts in habitat use in response to rainfall, temperature and water availability [24,25,26,27], as well as their use of agricultural areas [28,29,30,31,32,33,34,35]. In Uganda and Kenya, cranes move to wetlands during the breeding season [25,35,36]. In Tanzania, seasonal changes in human land use, including rice, millet and sorghum farming, also influence crane movements [37]. However, these studies are mainly based on observational data and did not use telemetry to directly track crane movement patterns.

We use GPS-GSM telemetry to investigate the movement ecology of Grey Crowned Cranes in Rwanda. Specifically, our goals are as follows: (1) characterize post-release movements of reintroduced cranes rescued from captivity, including dispersal and home range; (2) compare movement patterns between reintroduced and wild cranes; (3) identify environmental and management-related factors influencing crane movement. Given the provision of supplemental food at the release site, we hypothesized that reintroduced cranes would exhibit higher site fidelity, shorter dispersal distances and smaller home ranges compared with wild individuals. We further predicted that both groups would respond similarly to seasonal changes in precipitation and habitat availability.

## 2. Materials and Methods

### 2.1. Study Area

**Figure 1:**
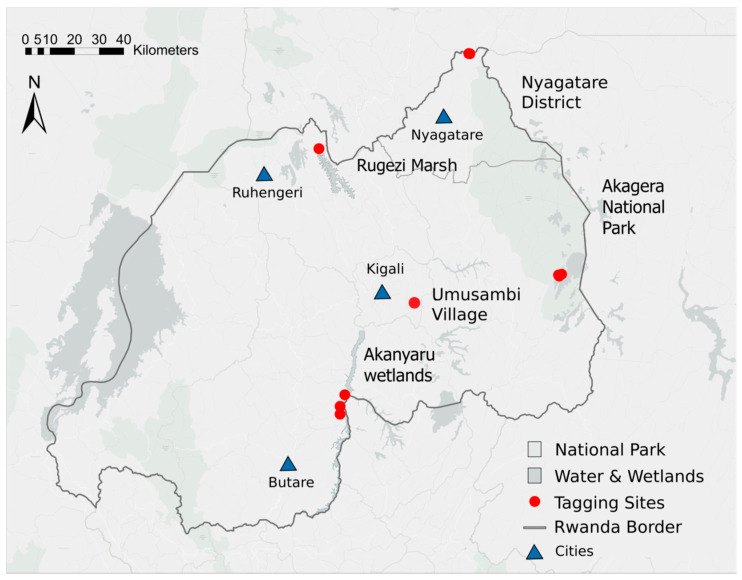
Geographic locations where Grey Crowned Cranes were fitted with GPS-GSM transmitters across Rwanda between 2019 and 2022. All reintroduced cranes were tagged at Akagera National Park. Only wild cranes were tagged at the other sites.

This study was conducted in five regions of Rwanda (Figure 1), selected based on Grey Crowned Crane presence and their relevance to conservation efforts. Three sites—Rugezi Marsh, Nyagatare, and Akanyaru Wetlands—were identified by the RWCA as areas with high crane occurrence based on multiple years of country-wide surveys conducted during the non-breeding season. Akagera National Park (NP) was included as the release site for all reintroduced cranes due to its suitable habitat for Grey Crowned Cranes, comparatively strong law enforcement, and effective logistical support through collaboration with the Akagera Management Company and Rwanda Development Board, which co-manage the park. Umusambi Village, located near Kigali, served as a sanctuary for rescued cranes that could not be released back into the wild.

Rugezi Marsh, located in northern Rwanda at 2100 m elevation, is a 6735 ha protected peat bog within the Buberuka Highlands. Recognized as an Important Bird Area (IBA) by BirdLife International since 2001, it supports one of the largest known populations of Grey Crowned Cranes in the country [38,39]. Nyagatare, in the northeast along the Muvumba River near the Ugandan border, includes the Matimba and Musheri sectors, which host crane flocks. The landscape is predominantly agricultural, with crops such as maize, beans, tomatoes, and soybeans. Akagera NP, in eastern Rwanda along the Tanzanian border, features savannah and wetland habitats. Over one-third of the park comprises lakes and interconnected papyrus swamps *Cyperus papyrus*. The park served as the sole release site for reintroduced cranes. Akanyaru Wetland, in southern Rwanda along the Burundi border, is an unprotected area dominated by marsh and papyrus vegetation. It plays a key role in regional crane ecology despite lacking formal protection. Umusambi Village is a 25-ha restored wetland and nature reserve located in Kigali. It functions as both a rehabilitation site and a permanent sanctuary for non-releasable cranes. Additional details of these study sites are provided by Nsengimana and Becker [39].

Rwanda’s high elevation contributes to its temperate tropical climate, with relatively stable temperatures ranging from 15 °C to 27 °C throughout the year. The country experiences four distinct rainfall-based seasons: a long rainy season from March to May, a short rainy season from September to December, as well as two dry seasons from June to August and January to February [40]. While these seasonal patterns are well established, regional and interannual rainfall variability is common [41]. Rainfall patterns observed during our study period were generally consistent with the typical seasonal classifications.

### 2.2. Crane Rescue, Rehabilitation, and Reintroduction

The ten reintroduced cranes tagged in this study were originally obtained as chicks and juveniles illegally captured in the wild. For nine of these individuals, the duration of captivity before rescue was known, ranging from 2 days to 10 years (Table 1). After their rescue, all cranes underwent a comprehensive physical examination followed by a 60-day quarantine at the RWCA facility. Upon completing quarantine, the rescued cranes were transferred directly to a soft-release enclosure in Akagera NP. 

At Akagera NP, cranes were soft-released into a 4.2 ha open-air enclosure situated along the shore of Lac Ihema. The enclosure was secured on three sides with electric fencing, while the lake-facing side remained open, allowing the birds to leave voluntarily. Despite this open design, most cranes remained within the enclosure during the initial period, as their flight feathers were still regrowing, and flight capacity was redeveloping following wing clipping during captivity. Supplemental food (Mazuri^®^ commercial bird feed produced by Mazuri Exotic Animal Nutrition, St. Louis, MO, USA) was continuously provided within the enclosure throughout the reintroduction program, from January 2015 to July 2021, after the last crane had left the enclosure. Supplemental food was reduced during the wet season when natural food sources, such as insects, were more abundant. As the cranes gradually dispersed from the enclosure and increased their reliance on natural foraging, supplemental feeding was progressively reduced.

### 2.3. Crane Capture and Tagging

A total of 21 Grey Crowned Cranes were trapped and tagged with GPS-GSM units at five locations in Rwanda between 2019 and 2022 (Figure 1), including 10 reintroduced individuals (designated “R”) and 11 wild individuals (“W”) (Table 1). For nine of the ten reintroduced cranes, the month of release into the enclosure was recorded. These individuals spent an average of 20 mos (SD = 9) in the enclosure prior to tagging (Table 1). The sex of tagged cranes was known for 7 of the 10 reintroduced cranes and only 2 of the wild cranes (Table 1).

All reintroduced cranes were trapped and tagged within the release enclosure at Akagera NP with tagging candidates selected on the following criteria: (1) absence of visible wing injuries or deformities, (2) potential for full regrowth of flight feathers, and (3) demonstrated flight capability within the enclosure. All reintroduced cranes were tagged as adults, except for R7 (~14 mos) and R8 (~16 mos). It is not known if any of these tagged cranes were family members. Wild cranes were tagged at four additional locations. Nine adult cranes were captured in Rugezi Marsh, Nyagatare, and Akanyaru Wetlands to assess movement patterns and habitat use within these regions. We suspect that the two cranes tagged at Rugezi Marsh were a mated pair based on their subsequent movements, while the relationships of all other tagged adult cranes were unknown. The remaining two wild-tagged cranes, W11 and W22, were trapped at Umusambi Village. Both were fledged chicks aged 10–14 weeks and had been raised by different adult pairs that included rescued, flightless individuals. W22 was reared by a pair of permanently disabled, rescued cranes, while W11 was raised by a mixed pair consisting of a wild male and a rescued, flightless female. All cranes were tagged during the non-breeding season to minimize potential disturbance during the breeding season.

All tagged cranes were captured using a toe-snare technique with monofilament snares, following the method described by Hereford & Dedrickson [11]. Individual snares were attached to sticks and placed in areas frequented by cranes for feeding, resting, and drinking. For capture of the reintroduced cranes within the enclosure, trap sites were pre-baited with corn and Mazuri^®^ commercial bird feed to increase capture success. Once a crane was snared, it was quickly retrieved using a run-and-catch method. A hood was placed over the bird’s head to reduce visual stimuli and minimize stress during handling. Each crane was examined to ensure it was in suitable physical condition before tagging. GPS-GSM units (Ecotone^®^, model Stork-H, 29 g, Ecotone Telemetry, os. Bernadowo 1D, Gdynia, Poland) were attached above the knee of the right leg (Figure S1) of 19 cranes and back-mounted units were used for the two cranes tagged at Rugezi Wetland (W18, W20). After tagging, the hood was removed, and the crane was released. Tags recorded GPS coordinates at variable intervals, with median fix rates ranging from 15 min to 6 h, depending on the individual crane.

Tracking duration varied widely among the 21 tagged cranes, ranging from 5 to 1118 days (Table 1). Reintroduced cranes had a median of 1722 recorded GPS locations (interquartile range [IQR]: 128–7216), while wild cranes had a median of 1311 locations (IQR: 96–3276). There was no statistically significant difference in the number of recorded locations between reintroduced and wild individuals (z = 0.46, *p* = 0.65).

The cause of tracking cessation was confirmed for only three reintroduced cranes. Crane R3 was found deceased under unknown circumstances; crane R8 was recaptured due to a toe injury, and the unit was removed for use on another crane. Crane R21 was killed by a leopard *Panthera pardus* within the release enclosure. As of 23 March 2025, GPS-GSM tags remained active for two cranes (W18 and W20). For all remaining individuals, tags ceased transmitting, and survival status is unknown.

Due to intermittent unit failure, two cranes (R8 and W22) experienced data gaps exceeding 100 days, resulting in limited usable location data. Additionally, four cranes (R2, R6, W13, and W14) were tracked for fewer than 20 days and were excluded from movement analyses due to insufficient data. However, crane W13 exhibited a notable long-distance dispersal and was therefore retained for inclusion in the dispersal analysis.

### 2.4. Dispersal

Net displacement—the straight-line distance from each location to the crane’s release site—was calculated using R version 4.5.0 [42] and the amt package 0.2.2.0 [43]. To model dispersal, we used one location per day to reflect broader, long-term movement patterns, following the method recommended by Bunnefeld et al. [44]. However, for calculating the maximum distance moved from the release site, we used the full dataset rather than the daily subset, because the most distant location was not always included in the one-point-per-day sample.

We defined dispersal as the movement of a crane that exceeded 10 km from its release site, with more than 50% of its subsequent net displacement occurring at or beyond that distance. We chose this threshold to distinguish true dispersal events from short-term exploratory movements. For example, crane R7 traveled more than 10 km from the release site but returned within eight days and did not leave the area again; we classified this movement as an exploratory foray rather than a dispersal event.

Because no published data exist on daily movement distances for Grey Crowned Cranes, the 10 km threshold was informed by studies of other crane species. For instance, Whooping Cranes typically moved an average of 4.12 km per day on non-travel days [45]. Studies on Greater *Grus canadensis tabida* and Lesser *G. c. canadensis* Sandhill Cranes reported that most daily commuting flights were within 5 km and 10 km of the roost site, respectively [46]. Similarly, Common Cranes exhibited maximum daily displacements of 6.5 km [47]. Based on these benchmarks for other crane species, we defined movements ≥ 10 km by Grey Crowned Cranes as exceeding the typical daily movement range, thereby constituting a practical threshold for identifying dispersal events.

To determine the timing of dispersal, we visually examined plots of net displacement from the release site over time (Figure S2), following methods described by Jahn et al. and Phipps et al. [48,49]. For abrupt dispersal events, we defined the start date as the first date a crane moved beyond 10 km. For gradual dispersals, the start date was the point at which net displacement began increasing continuously.

### 2.5. Home Ranges

We estimated home ranges using two methods: dynamic Brownian Bridge Movement Models (dBBMMs) and Kernel Density Estimations (KDEs). KDEs are widely used in ecological research [50], but dBBMMs offer several advantages when analyzing movement data from wide-ranging species such as cranes. Unlike KDEs, which assume temporally independent locations, dBBMMs account for both the time intervals between successive locations and the estimated movement paths [51]. Additionally, dBBMMs extend the traditional Brownian Bridge approach by dynamically estimating the Brownian motion variance (σ^2^_m_) along a sliding window. This process allows the model to reflect changes in movement behavior over time, rather than assuming a constant variance throughout the track [52]. dBBMMs are particularly effective for detecting behavioral shifts and quantifying space use in species capable of rapid or long-distance movements [53,54]. Despite these advantages, we also calculated KDEs to facilitate comparisons with other studies that employed more traditional methods.

We performed all analyses in R version 4.5.0 [42], using the move package 4.2.6 for dBBMMs [55]. We set the grid resolution to 25 × 25 m and the extent to 1. The location error was fixed to 9 m based on the manufacturer’s specifications for the GPS-GSM unit. The default window size was set to a 6-day interval, with a 2-day margin, based on the expected interval between behavioral changes [52]. However, due to variability in the time intervals between GPS locations, we allowed the window size to vary by individual track. We assumed a minimum plausible interval of 7 days between behavioral changes to define a biologically meaningful window size.

The smallest window size used was 23 locations, corresponding to individuals with a median sampling interval of 6 h. The largest window size—575 locations—was used for two individuals (W11 and W20) tracked with 15 min intervals. Although this window was substantially larger than the smallest, it was necessary to ensure accurate estimation of σ^2^_m_ for these two cranes. When we tested a window size ten times smaller, the resulting utilization distributions were overly inflated, indicating reduced estimation reliability.

To address data gaps, we removed σ^2^_m_ segments corresponding to time gaps > 24 h for several cranes (R1, R5, R7, W10, W15, W18, W20), following the recommendations in Smolla et al. [55]. We calculated KDEs using the adehabitatHR package 0.4.2.2 [56], with the reference bandwidth (href) as the smoothing parameter. As with dBBMMs, we used a 25 × 25 m grid extended by a value of 1. For both estimators, we extracted the 50% and 95% utilization distribution contours to represent core use areas and home ranges, respectively, for each crane.

### 2.6. Statistical Analysis

All statistical analyses were conducted in R (version 4.5.0) [42]. Due to substantial variation in movement patterns among individual cranes, we report medians and interquartile ranges rather than means. To compare maximum net displacement, time spent at the release site after tagging before dispersing, and home range size between reintroduced and wild cranes, we used Wilcoxon Rank-Sum Tests. We applied Kruskal–Wallis H Tests to assess whether home range size and maximum net displacement differed by release location. To evaluate the relationships between tracking duration and both maximum net displacement and home range size, we used Spearman’s rank correlation.

## 3. Results

### 3.1. Dispersal

Dispersal behavior varied substantially among individual cranes, with considerable differences in dispersal timing and distance. During the tracking period, only 2 of 7 reintroduced cranes dispersed, compared to 7 of 9 wild cranes (Table 2). The two reintroduced individuals, both adults, dispersed after 399 and 446 days at the release site post-tagging, whereas the median time to dispersal among the seven wild cranes (6 adults and 1 juvenile (W11)) was 101 days (IQR: 54–159) (Table 2). Despite this apparent difference, the small sample size limited statistical power, and the difference in time to dispersal after tagging between reintroduced and wild cranes was not statistically significant (W = 12, z = 2, *p* = 0.07).

**Figure 2:**
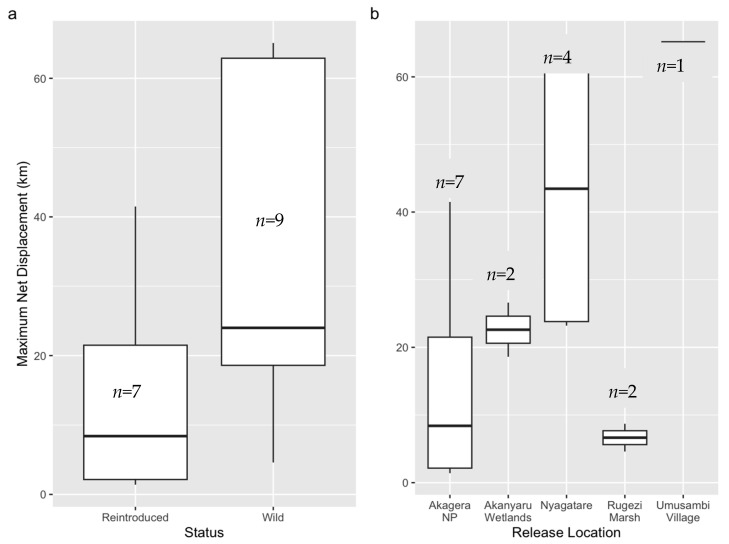
Maximum net displacement of Grey Crowned Cranes from their release sites. Straight-line distance from the release site for (**a**) reintroduced captive-rescued versus wild cranes, and (**b**) across different release locations (Table 3). The center line represents the median, the box spans the interquartile range (IQR), whiskers extend to the most extreme values within 1.5 × IQR from the quartiles, and *n* is the sample size.

There was considerable variation in the maximum distances cranes moved from their release sites, ranging from 1.4 km to 65.1 km (Table 3). Reintroduced cranes generally exhibited shorter maximum net displacements (Mdn = 8.4 km, IQR: 2.2–21.5) than wild cranes (Mdn = 24.0 km, IQR: 18.6–62.9) (Figure 2a). However, this difference was not statistically significant (W = 14, z = −1.85, *p* = 0.07), likely due to high variance and limited sample size.

Maximum net displacement also varied by release location (Figure 2b). All wild cranes tagged and released at Nyagatare, Umusambi Village, and the Akanyaru Wetlands traveled at least 18 km from their release sites. In contrast, the two wild cranes released in Rugezi Marsh remained within the marsh throughout the monitoring period of over 1000 days, exhibiting a mean maximum displacement of only 6.7 km (SD = 2.9) (Table 3). Notably, two wild cranes tagged at Nyagatare (W12, W13) dispersed over 60 km within 47 and 4 days of tagging, respectively, and did not return to their tagging sites. Although maximum displacement varied among release sites, these differences were not statistically significant (H(4) = 7.85, *p* = 0.10), again likely due to small sample sizes and high variability.

Maximum net displacement was strongly correlated with the length of the tracking period for both reintroduced (r = 0.84, *p* = 0.02) and wild cranes (r = −0.69, *p* = 0.04), but the correlations were in opposite directions. As a result, no significant relationship was found between tracking duration and displacement when combining reintroduced and wild cranes (r = −0.14, *p* = 0.59).

Among the five reintroduced cranes that did not disperse, all frequently exited the enclosure but remained in the vicinity of the release site (Figure S3). Most moved a maximum of 1.4–8.4 km, although one individual (R7) made a longer movement of 16.9 km (Table 3). We considered these movements exploratory forays, and the net displacement of these five non-dispersing cranes over time are shown in Figure S4, starting at the release enclosure at Akagera NP.

Of the nine cranes that dispersed (2 reintroduced and 7 wild), most (*n* = 8) cranes did not return to their release site after dispersal, except for R5 (Figure S2). At 446 days after tagging, this crane dispersed 25 km from its release site at Akagera NP and regularly returned to its release site over a 6 mo period (September 2020–February 2021). This crane returned to its release site 28 times, once every 1–19 days, remaining there for only a short time (M = 24 h, SD = 32). Subsequently, this crane relocated to a new site 10 km from the release site. Another reintroduced crane (R7) made a single movement of 16.9 km from the Akagera NP release site, but returned after a week, suggesting that this movement was an exploratory foray rather than a dispersal.

Considering that all tagged cranes were released within 10 km of an international border (except W11 and W22 tagged at Umusambi Village), several cranes made international transboundary movements during dispersal and exploratory movements. All four wild cranes released in Nyagatare dispersed over Rwanda’s northern border into Uganda. Reintroduced cranes R1, R5, and R7 at Akagera NP moved across the eastern border into Tanzania, although crane R5 only had one recorded movement of 0.1 km over the border. In the Akanyaru Wetland, wild cranes W10 and W16 made forays into Burundi, but never moved more than 0.1 km across this southern border.

### 3.2. Home Range

**Figure 3:**
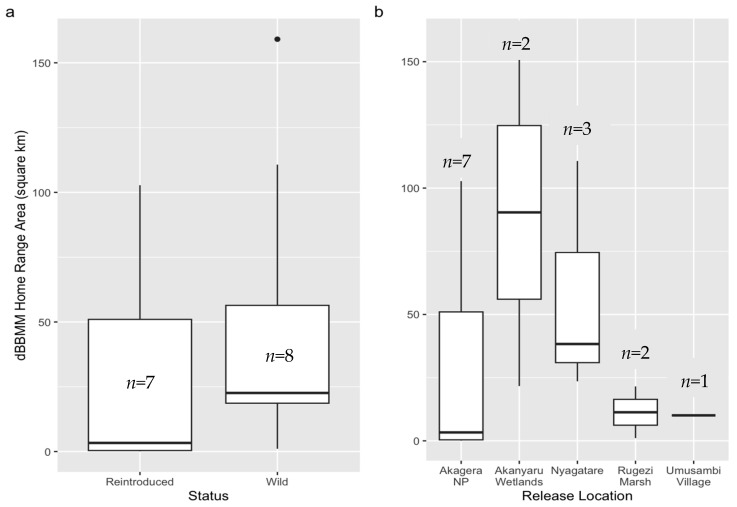
Home range sizes of Grey Crowned Cranes estimated using dBBMM utilization distributions. Boxplots of home range sizes derived from 95% dynamic Brownian Bridge Movement Model (dBBMM) utilization distributions for (**a**) reintroduced captive-rescued versus wild cranes, and (**b**) across different release locations (Table 4). The center line represents the median, the box spans the interquartile range (IQR), whiskers extend to the most extreme values within 1.5 × IQR from the quartiles, and *n* is the sample size.

There was substantial variation in home range sizes (95% dBBMM) among both reintroduced and wild cranes, as well as across the five release locations (Table 4). Although reintroduced cranes had a notably smaller median home range (Mdn = 3.3 km^2^, IQR: 0.4–51.0) compared to wild cranes (Mdn = 22.6 km^2^, IQR: 18.7–56.4) (Figure 3a), this difference was not statistically significant (W = 15, z = −1.5, *p* = 0.15), likely due to high individual variability and limited sample sizes. Similarly, while home range sizes varied across release sites (Figure 3b), these differences were also not significant (H(4) = 5.42, *p* = 0.25), again reflecting the effects of high variance and small sample size. Representative home-range plots are provided in Figure S5 for the dBBMM estimates.

Given the substantial variation in tracking durations among individuals (Table 1), we examined the relationship between tracking period length and home range size. Although home range size tended to increase with tracking duration in reintroduced cranes, the correlation was not statistically significant (Spearman’s r = 0.75, *p* = 0.066), probably due to the small sample size. No significant correlation was found for wild cranes (r = −0.11, *p* = 0.84), nor when both reintroduced and wild cranes were analyzed together (r = 0.28, *p* = 0.32).

## 4. Discussion

The reduced dispersal behavior observed in reintroduced Grey Crowned Cranes—evidenced by fewer dispersing individuals, longer time intervals between tagging and dispersal, and shorter maximum net displacements—supports our hypotheses that reintroduced cranes would exhibit greater site fidelity and shorter dispersal distances compared to their wild counterparts. Notably, only 2 of 7 reintroduced cranes dispersed during the study period, compared to 7 of 9 wild cranes.

A likely explanation for this pattern is the long-term provision of supplemental food at the release enclosure in Akagera NP, which continued throughout the crane reintroduction program. This consistent food source likely reduced the need or motivation for cranes to disperse or establish new home ranges. Food provisioning ceased only after the last crane left the enclosure in July 2021—well after most reintroduced individuals (*n* = 166) had spent extended periods at the site. The combination of a reliable food supply and environmental familiarity likely contributed to the cranes’ continued use of the enclosure and their limited dispersal. Davis [8] noted that artificial feeding can lead to substantial increases in small crane populations but also cautioned that it should be considered a short-term strategy for population support.

In addition to the effects of supplemental feeding, the capture of cranes as chicks and their extended periods of captivity before rescue may have influenced the limited dispersal observed in reintroduced cranes. All but two (R7 and R8) of the nine reintroduced individuals had been held in captivity for prolonged periods of 4 to 10 years, which probably reduced their propensity to disperse. Notably, even R7 and R8—who had relatively short captivity histories (2 days and 4 mos, respectively)—remained in or near the enclosure for extended periods (13 and 10 mos, respectively) before they were tagged in the enclosure. This further suggests that the availability of supplemental food played a significant role in delaying movement away from the enclosure. In addition, reintroduced cranes required time to regain full flight capability following captivity. This included the complete regrowth of flight feathers, which could extend up to a year or more, and the redevelopment of flight muscles—factors that likely contributed to delayed or reduced dispersal or movements away from the enclosure. Similar patterns have been documented in soft releases of other species, where individuals often exhibit reduced movement and increased site fidelity post-release [57,58].

This reduced tendency to disperse from the enclosure was reflected in the home range sizes of reintroduced cranes. Reintroduced individuals had substantially smaller home ranges (95% dBBMM) than wild cranes (Figure 3a), consistent with their more sedentary behavior. Again, the extended provision of supplemental food at the release site likely minimized the need for wide-ranging movements in search of resources.

In the absence of a reliable and abundant supplemental food source, we hypothesized that tagged wild cranes would exhibit lower site fidelity, greater dispersal distances, and larger home ranges compared to reintroduced individuals. Overall, our findings support this hypothesis: 7 of the 9 wild cranes dispersed, traveled farther from their tagging locations, and occupied larger home ranges. However, considerable variation in crane movement behavior was observed both between tagging locations and among individual cranes tagged within the same location.

For instance, the two wild cranes tagged at Rugezi Marsh did not disperse, remaining within <9 km of their tagging site. These individuals were tagged on the same day at nearby locations (within 24 m), and their home ranges showed substantial overlap over nearly three years of tracking, suggesting they were probably a mated pair. In contrast, all four wild cranes tagged at Nyagatare dispersed over variable distances. Among them, W12 and W13 dispersed over 60 km to the same general location. Despite this shared endpoint, the two birds dispersed independently, departing 41 and 4 days after tagging, respectively (Table 2, Figure S2). Further, both units stopped transmitting within seven days of initiating dispersal, raising the possibility that the two individual cranes may have died following these relatively long dispersals. The remaining two cranes at Nyagatare, W15 and W17, also dispersed independently, departing approximately three months apart (Table 2) and moving to different destinations (Figure S5). These patterns highlight the diversity of dispersal behavior among wild cranes and the potential influence of site-specific and individual-level factors on movement outcomes.

Previous research reported that a variety of biological and environmental factors influence movement patterns in Grey Crowned Cranes. Meine and Archibald [59] characterized the species as opportunistic and nomadic, with food availability and nesting site quality as key determinants. In Uganda, Nachuha et al. [25] identified food availability, habitat quality, weather conditions, and predation pressure as major influences on crane distribution. Seasonal variation also plays an important role. Amulike et al. [24] reported that cranes typically breed during the rainy season, forming isolated pairs, and aggregate into larger foraging groups in the dry season in Tanzania. In Rwanda, Niyomwungeri [26], using a subset of the same individuals tracked in the present study, reported that home range size expanded during the short dry and long rainy seasons. He concluded that food distribution was the primary driver of larger home ranges, while breeding activity contributed to more localized movements. Additionally, he reported that international transboundary movements were more frequent during the dry season, with temperature identified as a key influencing factor.

While we hypothesized that seasonal variation would similarly affect crane dispersal in our study, our ability to detect such patterns was constrained by limited statistical power due to the small sample size and variation in tracking duration across individuals. Consequently, we were unable to robustly assess the relative influence of environmental and biological factors on dispersal or nesting behaviors.

### Study Limitations and Implications for Reintroduction Success

Several methodological constraints should be considered when interpreting the movement patterns of reintroduced and wild cranes described above. First, statistical power was limited by the small number of cranes with sufficiently long tracking periods. Nearly half of the individuals were tracked for less than one year, restricting our ability to evaluate behavior across complete annual cycles or to detect seasonal influences on dispersal. This limitation also prevented robust assessment of breeding or nesting behavior. Movement-based inference of reproduction requires extended, high-resolution tracks. Still, the combination of short tracking intervals, the restricted movements of most reintroduced cranes, and substantial variability among wild individuals provided insufficient evidence to reliably diagnose breeding status.

Second, transmitter failures created data gaps, reducing our ability to determine the exact dispersal timing for some cranes and their fates. Although battery diagnostics indicated that transmitter failures were not caused by low voltage, the final GPS positions could not distinguish between tag malfunction, detachment, or mortality. As a result, incomplete tracks were interpreted conservatively, potentially leading to an underestimation of dispersal events for both reintroduced and wild cranes.

Third, the absence of tagged wild cranes at Akagera NP limited our ability to separate the effects of reintroduction history from site-specific environmental conditions. Although wild cranes were tracked in other regions, heterogeneity in habitat structure and landscape context likely contributed to the observed individual variation, reducing the direct comparability of movement patterns between reintroduced and wild birds.

Despite these constraints, the study provides rare empirical evidence on the post-release behavior of cranes rescued from long-term captivity—a phase of reintroduction for which quantitative data remain scarce. These findings underscore the importance of integrating movement data with demographic metrics, such as survival and reproduction, to more fully evaluate reintroduction outcomes [60]. Future efforts should prioritize larger, more spatially balanced samples, longer, more uniform tracking durations, improved transmitter reliability, and coordinated monitoring of reintroduced and wild cranes within shared landscapes. Such enhancements will be essential for assessing long-term population viability and refining reintroduction strategies for Grey Crowned Cranes and other species undergoing similar conservation interventions.

## 5. Conclusions

This study offers the first quantitative evaluation of post-release movement behavior in rescued Grey Crowned Cranes reintroduced in Rwanda. Our results show distinct differences between reintroduced and wild birds. Reintroduced cranes exhibited limited dispersal, shorter maximum net displacements, and smaller home ranges, consistent with extended captivity before release, the time needed to regain flight fully, and ongoing supplemental feeding at the release site. In contrast, wild cranes dispersed more frequently and showed greater movement variability across study locations.

These patterns should be interpreted with caution due to several methodological constraints, including small sample sizes, uneven tracking durations, transmitter failures, and incomplete seasonal coverage. These limitations hindered our ability to assess seasonal effects, infer breeding behavior, or determine the fate of individuals. Additionally, the lack of tagged wild cranes at Akagera NP limits direct comparison between reintroduced and wild birds under the same environmental conditions.

Despite these challenges, the data provide vital baseline information on post-release movement behavior in a species lacking such data. The extended presence of reintroduced cranes at the release site underscores the importance of supplemental feeding and high-quality habitat during the initial reintroduction phase. Ongoing monitoring—especially of survival, reproduction, and long-term space use—is essential to assess whether reintroduced birds fully integrate into the wild population and contribute to its long-term viability. These insights can inform ongoing and future reintroduction efforts for Grey Crowned Cranes in Rwanda and other species with similar rescue and rehabilitation histories.

## Figures and Tables

**Table 1:** Summary of GPS-GSM tracking data for reintroduced captive-rescued and wild Grey Crowned Cranes monitored in Rwanda from 2019 to 2025. Information includes release location, sex (if known), tagging date, tracking duration, and number of locations recorded. For reintroduced cranes, additional details are provided on time in captivity prior to rescue, date of release into the enclosure, and time spent in the enclosure prior to tagging with GPS-GSM units.

Status & Tagging Location	GPS-GSM Tracking Number	ID	Sex	Time in Captivity (Yrs)	Date of Release into Enclosure	Time in Enclosure Before Tagging (mos)	Tagging Date	Age at Tagging	Tracking Duration (Days)	Number of Locations Recorded
Reintroduced cranes										
Akagera National Park	48536475213	R1	F	6	7 September 2017	19	17 April 2019	Ad ^a^	802	14,146
	48536466931	R2					2 July 2019	Ad	5	15
	48536020733	R3	M	10	7 September 2017	19	17 April 2019	Ad	267	1420
	48536436915	R4	M	10	March 2018	13	17 April 2019	Ad	428	2559
	48536904221	R5	F	8	16 March 2017	25	17 April 2019	Ad	735	10,806
	48536808827	R6	M	8	16 March 2017	39	9 June 2020	Ad	19	73
	48536464787	R7	M	2 days	March 2018	13	17 April 2019	~14 mos	435	8768
	48531069990	R8		120 days	11 July 2018	10	9 May 2019	~16 mos	403	32
	48535704486	R9	F	6	7 September 2017	20	8 May 2019	Ad	337	2024
	48536020733	R21		4	11 July 2018	26	6 September 2020	Ad	69	291
Wild cranes										
Akanyaru wetlands	48576271080	W10					6 May 2021	Ad	231	1311
	48575584891	W14					2 July 2020	Ad	11	41
	48575178409	W16					4 June 2020	Ad	427	1704
Umusambi Village	48575687341	W11					4 May 2021	~10 wks	415	886
	48531069990	W22					10 March 2021	~14 wks	221	23
Nyagatare	48576256437	W12	M				23 June 2020	Ad	48	151
	48575140289	W13					23 June 2020	Ad	5	12
	48575254578	W15	M				23 June 2020	Ad	1118	5255
	48575263799	W17					23 June 2020	Ad	463	1833
Rugezi Marsh	48533565131	W18					8 June 2022	Ad	1017	4719
	48575587474	W20					8 June 2022	Ad	1020	29,951

^a^ Ad = adult stage with Grey Cown Cranes obtaining adult plumage at ~3 years of age.

**Table 2:** Dispersal metrics for reintroduced captive-rescued and wild Grey Crowned Cranes released in Rwanda from 2019 to 2022. “Days since tagging” refers to the number of days between GPS-GSM tagging and the onset of dispersal.

Status	Release Location	ID	Dispersal Start Date	Days Since Tagging
Reintroduced	Akagera National Park	R1	20 May 2020	399
		R5	6 July 2020	446
Wild	Akanyaru Wetlands	W10 ^a^	11 November 2021	189
		W16 ^a^	23 September 2020	111
	Umusambi Village	W11 ^b^	–	–
	Nyagatare	W12 ^a^	3 August 2020	41
		W13 ^a^	27 June 2020	4
		W15	15 December 2020	175
		W17	23 September 2020	92

^a^ Tags on these cranes stopped functioning during dispersal; actual dispersal distances may therefore be underestimated. ^b^ Start date of dispersal could not be determined due to intermittent transmitter failure.

**Table 3:** The maximum net displacement (straight line distance) of reintroduced captive-rescued and wild Grey Crowned Cranes from their release site, as well as the number of days after GPS-GSM tagging that this maximum was reached.

Status	Release Location	ID	Maximum Net Displacement from Release Site (km)	Days Since Tagging
Reintroduced	Akagera National Park	R1	41.5	733
		R3	2.9	184
		R4	1.4	3
		R5	26.2	641
		R7	16.9	429
		R9	8.4	27
		R21	1.4	18
Wild	Akanyaru wetlands	W10	26.6	199
		W16	18.6	426
	Umusambi Village	W11	65.1	375
	Nyagatare	W12	62.9	47
		W13	62.9	4
		W15	24.0	849
		W17	23.2	354
	Rugezi Marsh	W18	4.6	7
		W20	8.7	440

**Table 4:** Home ranges and core areas (95% and 50% utilization distributions, respectively) of reintroduced captive-rescued and wild Grey Crowned Cranes estimated using Kernel Density Estimation (KDE) and dynamic Brownian Bridge Movement Models (dBBMMs).

Status	Release Location	ID	95% KDE Home Range (km^2^)	50% KDE Core Area (km^2^)	95% dBBMM Home Range (km^2^)	50% dBBMM Core Area (km^2^)
Reintroduced	Akagera National Park	1	337.03	37.83	87.39	3.01
		3	0.34	0.04	0.56	0.02
		4	0.06	0.01	0.09	0.01
		5	205.56	21.31	102.77	1.25
		7	21.02	3.36	14.62	1.49
		9	3.84	0.23	3.33	0.01
		21	0.14	0.03	0.24	0.02
Wild	Akanyaru wetlands	10	140.77	15.06	159.10	1.16
		16	18.03	2.89	21.67	0.54
	Umusambi Village	11	19.12	4.47	10.08	1.06
	Nyagatare	12	122.09	22.51	23.55	2.80
		15	146.98	24.72	110.71	9.86
		17	136.91	20.11	38.31	0.75
	Rugezi Marsh	18	0.67	0.12	1.08	0.11
		20	8.05	0.32	21.52	1.50

## Data Availability

Much of the data used in the analyses in this paper are given in the tables. The specific GPS locations of tagged cranes used in the analyses are available from the senior author, Deo Ruhagazi <deo@rwandawildlife.org>, upon request.

## Supplementary Materials

The following supporting information can be downloaded at: https://www.mdpi.com/article/10.3390/ani16010006/s1, Figure S1: Tagged Grey Crowned Crane with GPS-GSM unit (photo by Alex Cortellesi); Figure S2: Net displacement (km) from the release site by date for nine Grey Crowned Cranes that dispersed following GPS-GSM tagging. This includes two reintroduced captive-rescued individuals (R1 and R5) at Akagera National Park and seven wild individuals from three other locations. The first date on each trajectory represents the tagging date; Figure S3: Local movement patterns of five reintroduced Grey Crowned Cranes classified as non-dispersers, released at Akagera National Park. Boundary of the 4.2-ha release enclosure is shown in red. Tracking duration and number of locations for each individual are provided in Table 1. Longer exploratory movements by R7 and R9 are not depicted here but are detailed in Table 3 and Figure S4. Base map imagery: ESRI, DigitalGlobe, Earthstar Geographics, CNES/Airbus DS, USDA FSA, USGS, Aerogrid, IGN, IGP, and the GIS User Community; Figure S4: Net displacement (km) from the release site over time for seven Grey Crowned Cranes classified as non-dispersers and equipped with GPS-GSM transmitters. This group includes five reintroduced captive-rescued individuals (R3, R4, R7, R9, R21) released at Akagera National Park and two wild individuals (W18, W20) tagged at Rugezi Marsh. The first date on each trajectory marks the tagging date; Figure S5: Estimated home ranges (95% utilization distributions) of reintroduced captive-rescued and wild Grey Crowned Cranes in Rwanda, calculated using dynamic Brownian Bridge Movement Models (dBBMM). Grey points represent individual GPS locations, and red contours indicate the 95% utilization distribution boundaries. Reintroduced cranes were released at Akagera NP, while wild cranes were tagged at Akanyaru Wetlands, Umusambi Village, Nyagatare, and Rugezi Marsh. All panels are uniformly scaled (70 km × 70 km) and centered on a consistent reference point within each release area. Axes are measured in meters, and each white grid line represents a distance of 10 km.
