# Supplementary material for: Reintroduced Grey Crowned Cranes (Balearica regulorum) Exhibit Reduced Dispersal and Smaller Home Ranges than Wild Conspecifics in Rwanda
Source: Animals (Basel). 2025 Dec 19;16(1):6. doi: 10.3390/ani16010006 (PMC12785080; doi:10.3390/ani16010006)
Supplement: Supplementary file 1 [file animals-16-00006-s001.zip › animals-3977030-supplementary.pdf]

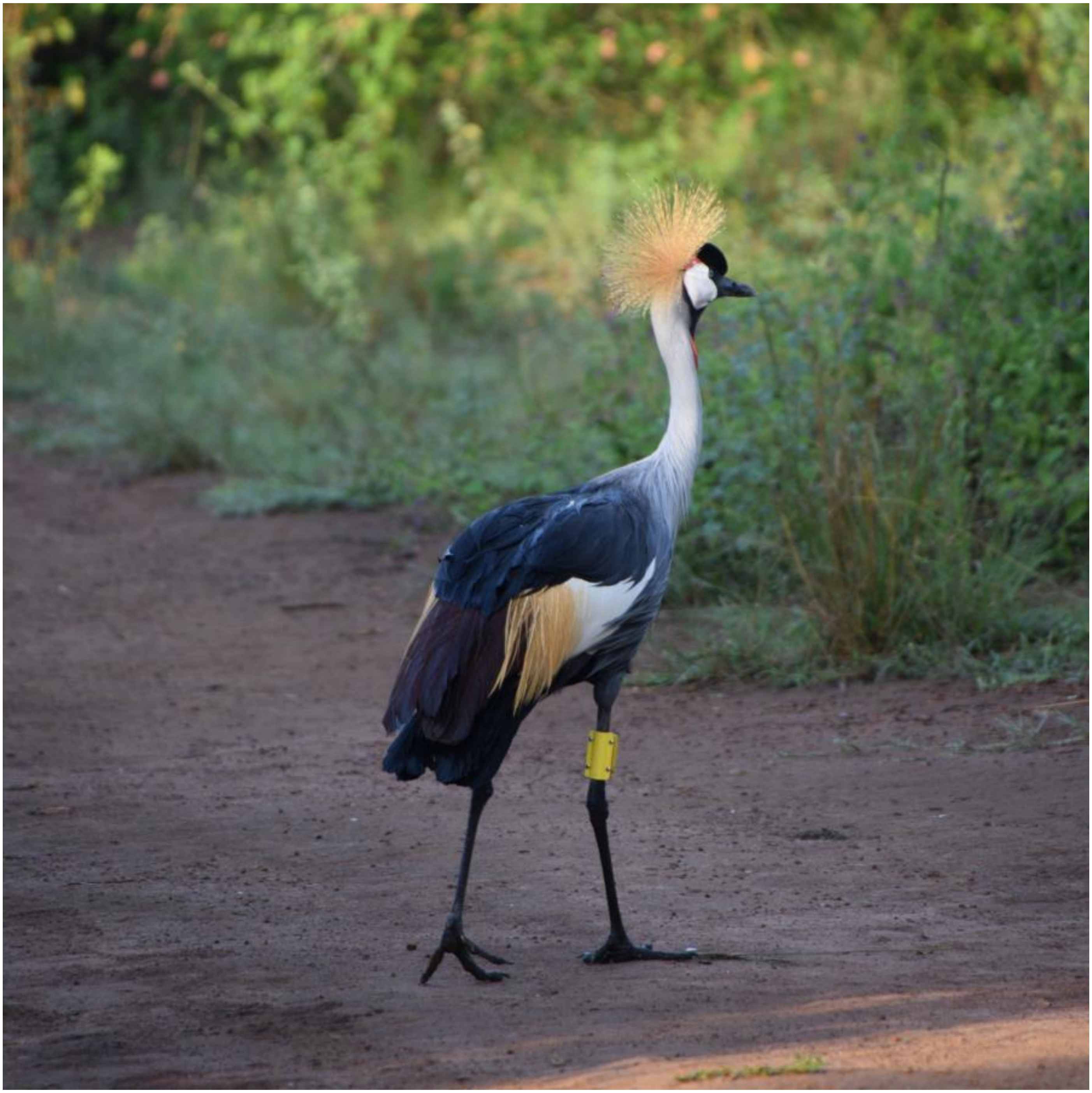

Figure S1. Tagged Grey Crowned Crane with GPS-GSM unit (photo by Alex Cortellesi).

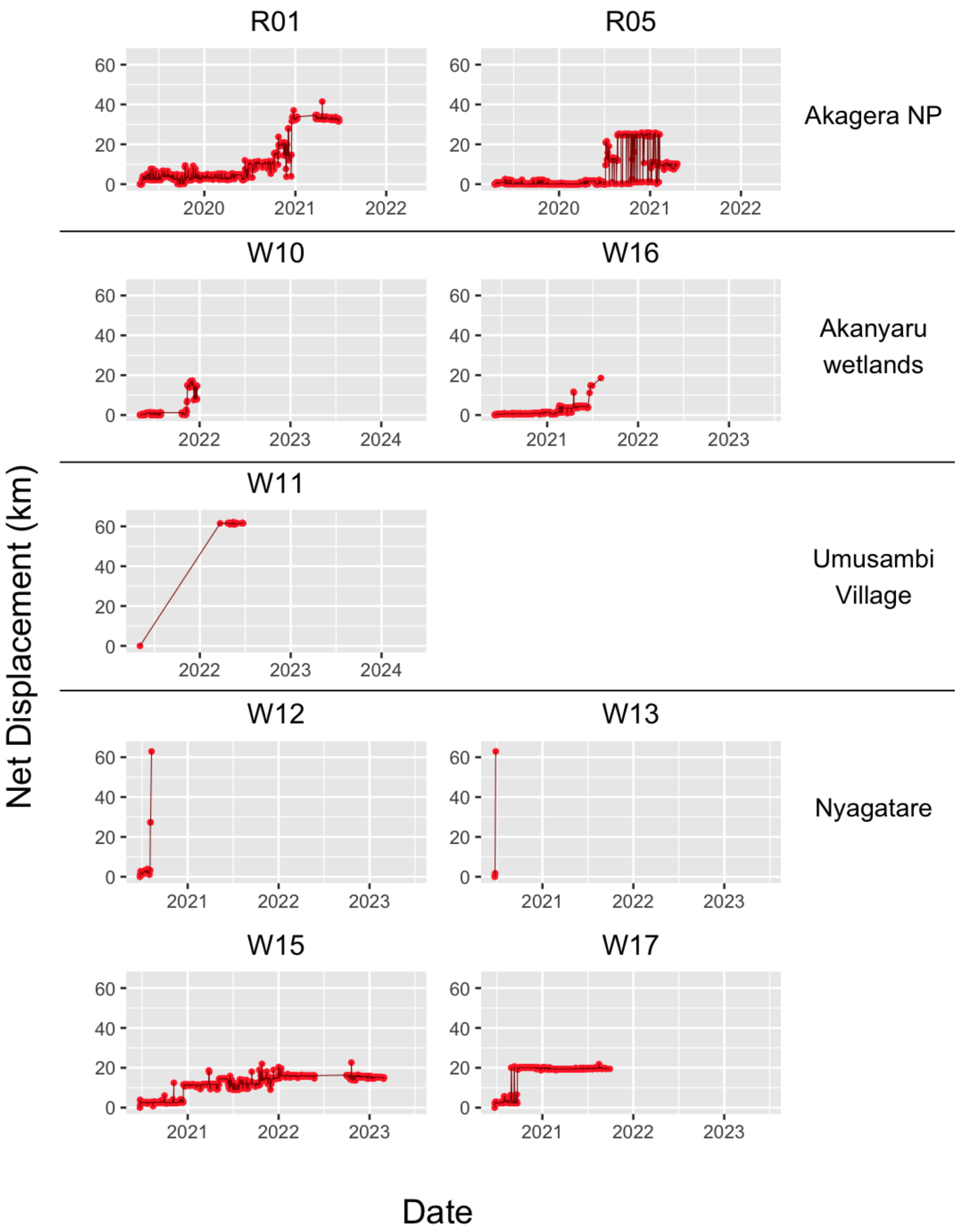

Figure S2. Net displacement (km) from the release site by date for nine Grey Crowned Cranes that dispersed following GPS-GSM tagging. This includes two reintroduced captive-rescued individuals (R1 and R5) at Akagera National Park and seven wild individuals from three other locations. The first date on each trajectory represents the tagging date.

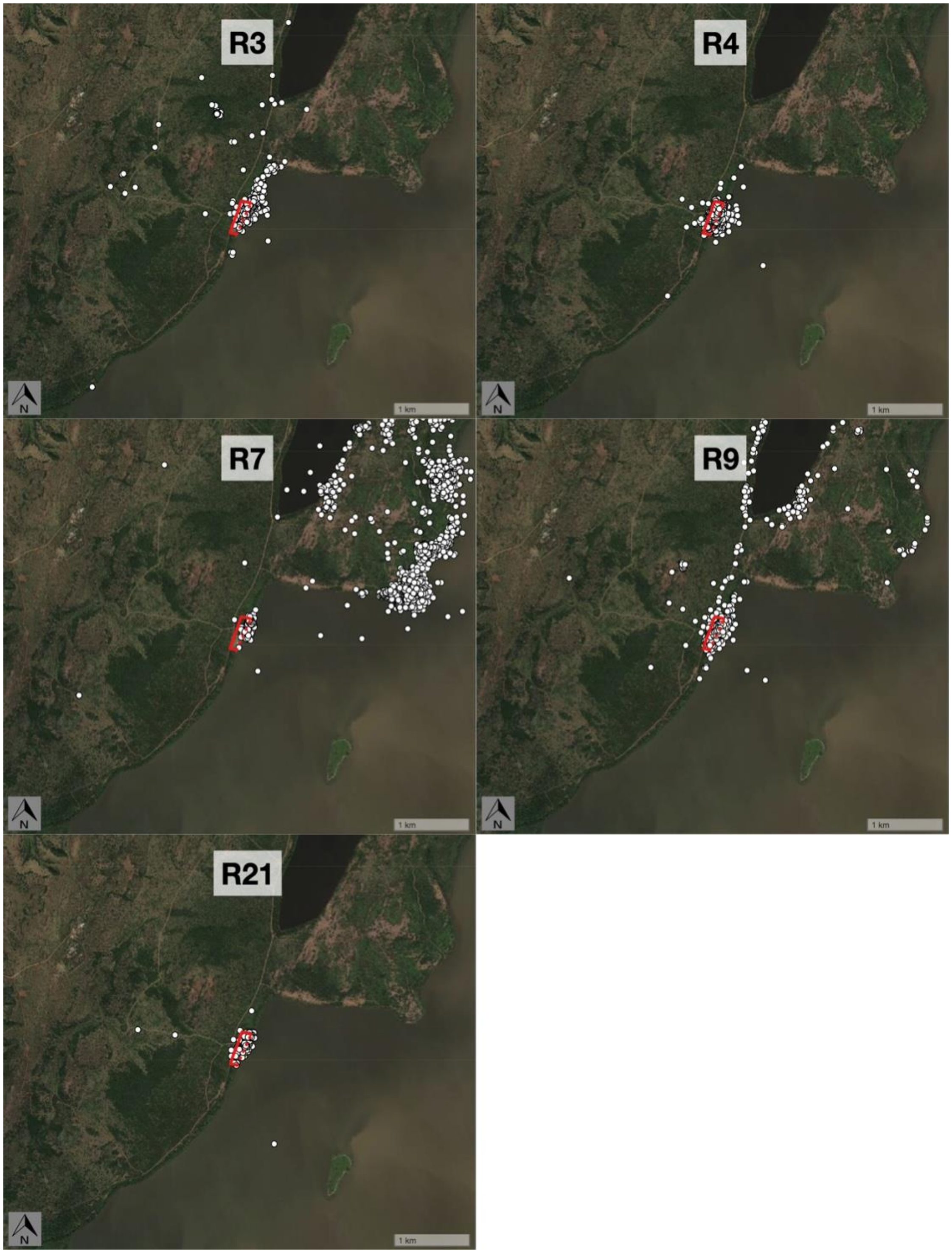

Figure S3. Local movement patterns of five reintroduced Grey Crowned Cranes classified as non-dispersers, released at Akagera National Park. Boundary of the 4.2-ha release enclosure is shown in red. Tracking duration and number of locations for each individual are provided in Table 1. Longer exploratory movements by R7 and R9 are not depicted here but are detailed in Table 3 and Figure S4. Base map

imagery: ESRI, DigitalGlobe, Earthstar Geographics, CNES/Airbus DS, USDA FSA, USGS, Aerogrid, IGN, IGP, and the GIS User Community.

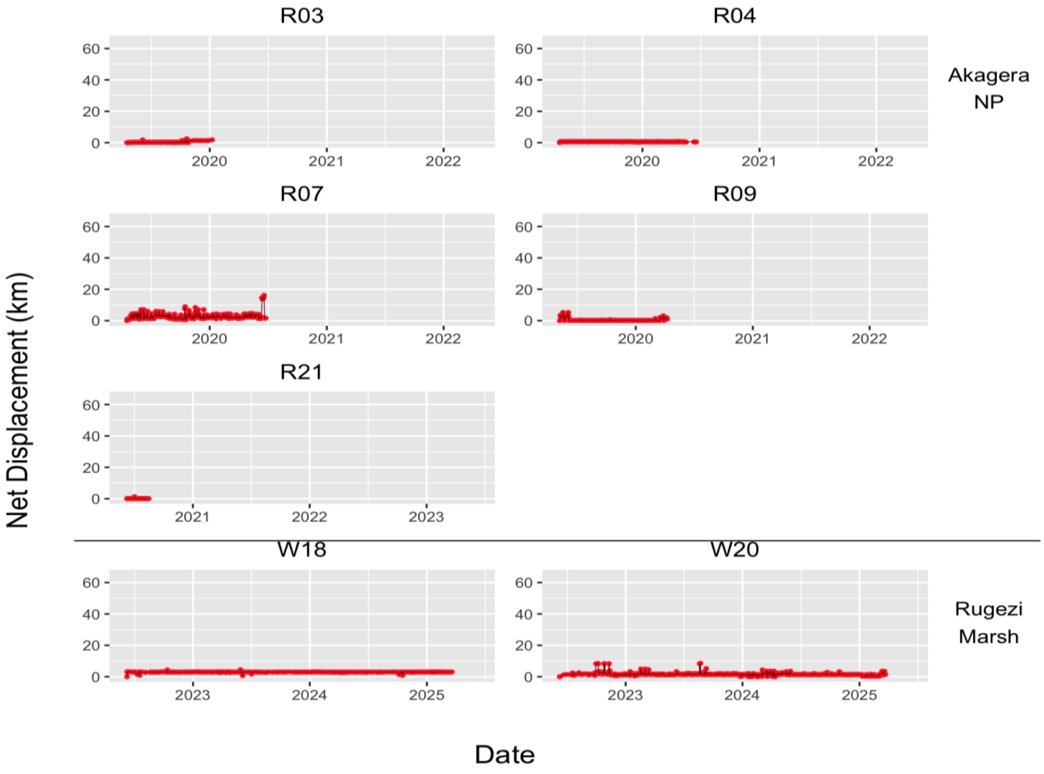

Figure S4. Net displacement (km) from the release site over time for seven Grey Crowned Cranes classified as non-dispersers and equipped with GPS-GSM transmitters. This group includes five reintroduced captive-rescued individuals (R3, R4, R7, R9, R21) released at Akagera National Park and two wild individuals (W18, W20) tagged at Rugezi Marsh. The first date on each trajectory marks the tagging date.

Akagera National Park

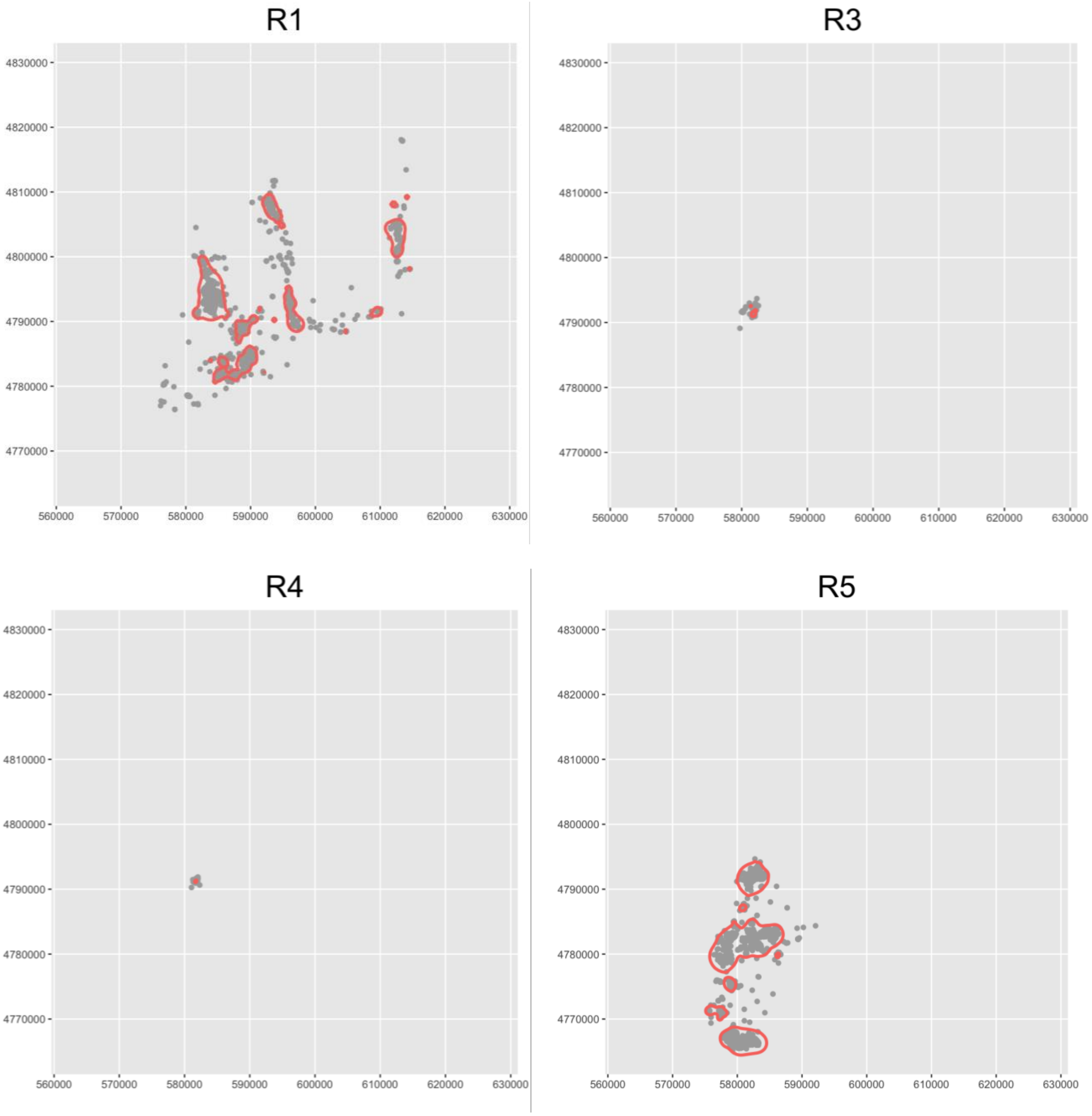

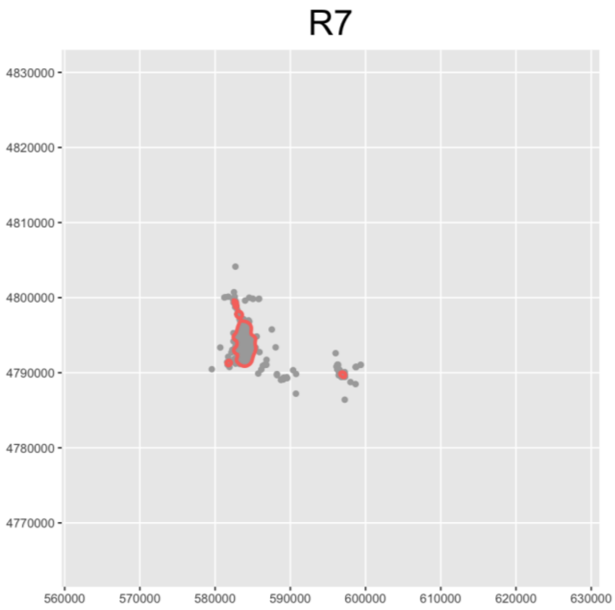
R7
4830000
4820000
4810000
4800000
4790000
4780000
4770000
560000
570000
580000
590000
600000
610000
620000
630000

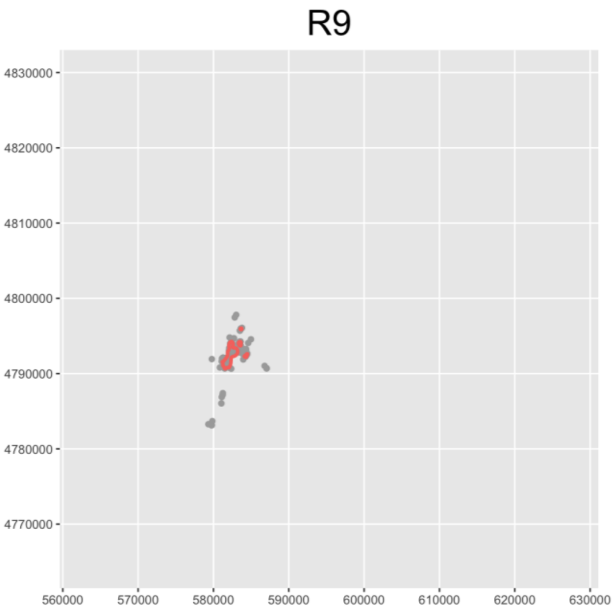
R9
4830000
4820000
4810000
4800000
4790000
4780000
4770000
560000
570000
580000
590000
600000
610000
620000
630000

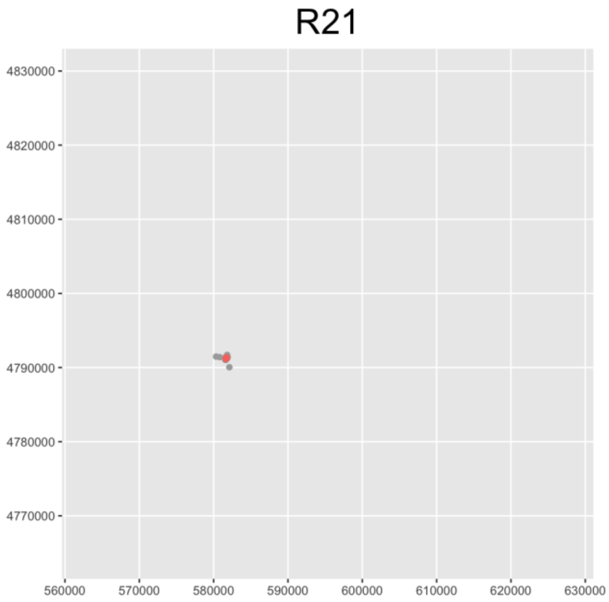
R21
4830000
4820000
4810000
4800000
4790000
4780000
4770000
560000
570000
580000
590000
600000
610000
620000
630000

Akanyaru Wetlands

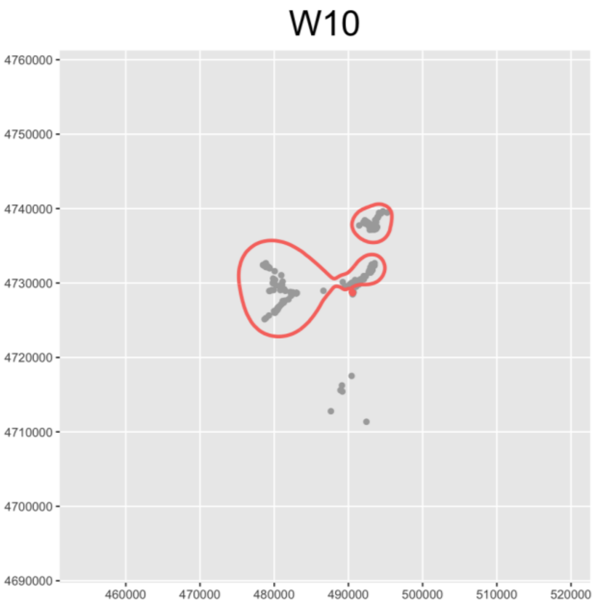

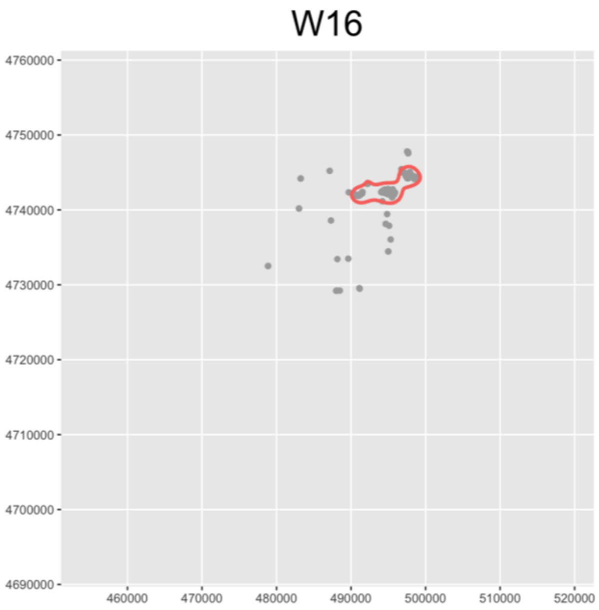

Umusambi Village

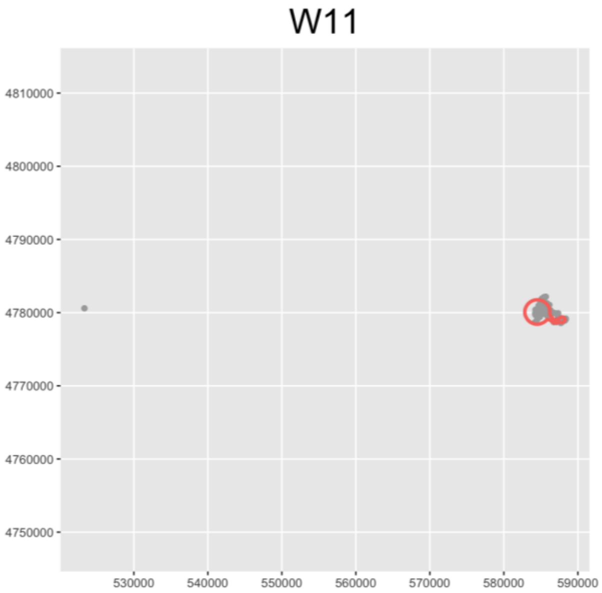

Nyagatare

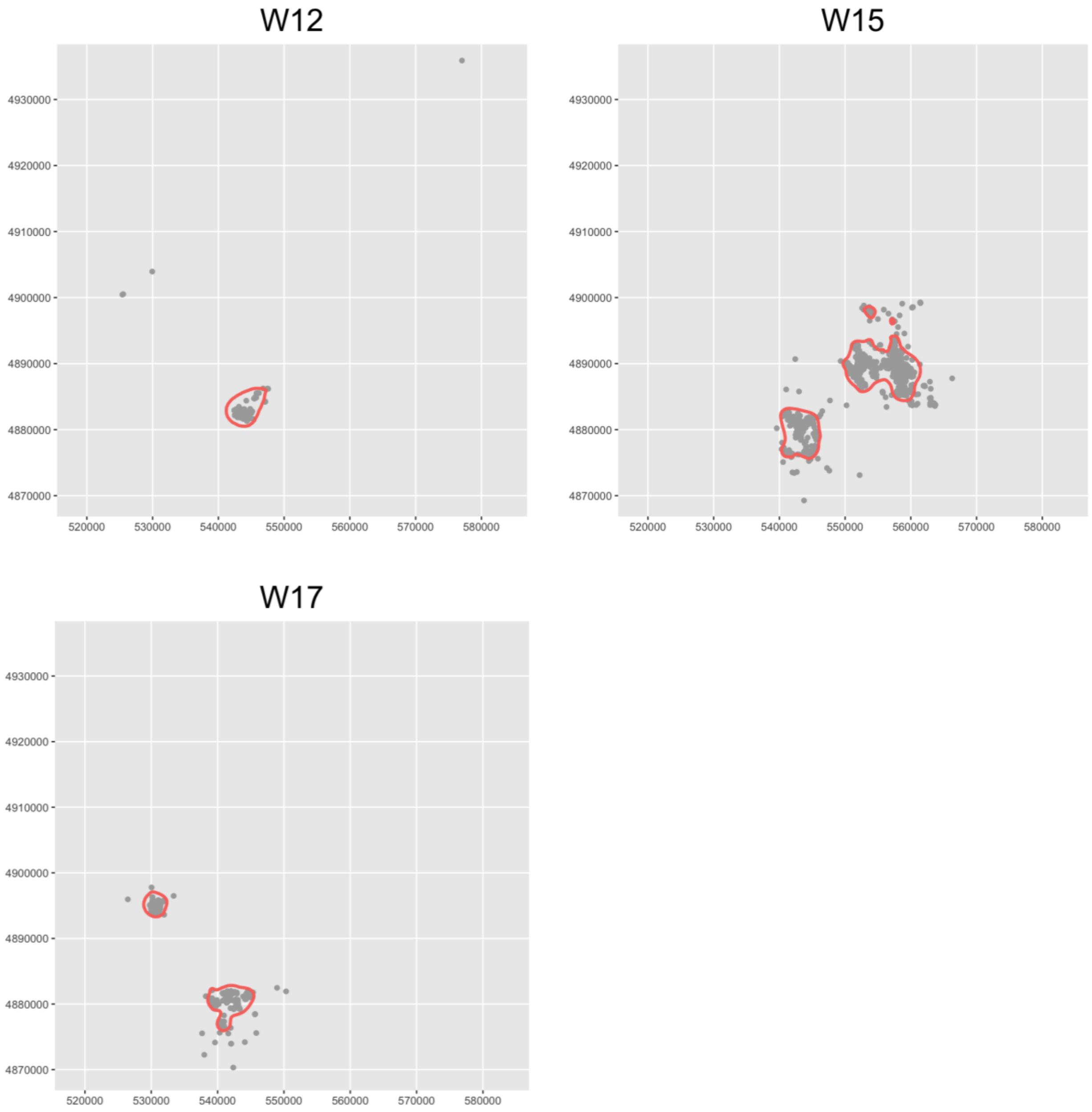
W12
W15
W17
4930000
4920000
4910000
4900000
4890000
4880000
4870000
520000
530000
540000
550000
560000
570000
580000

Rugezi Marsh

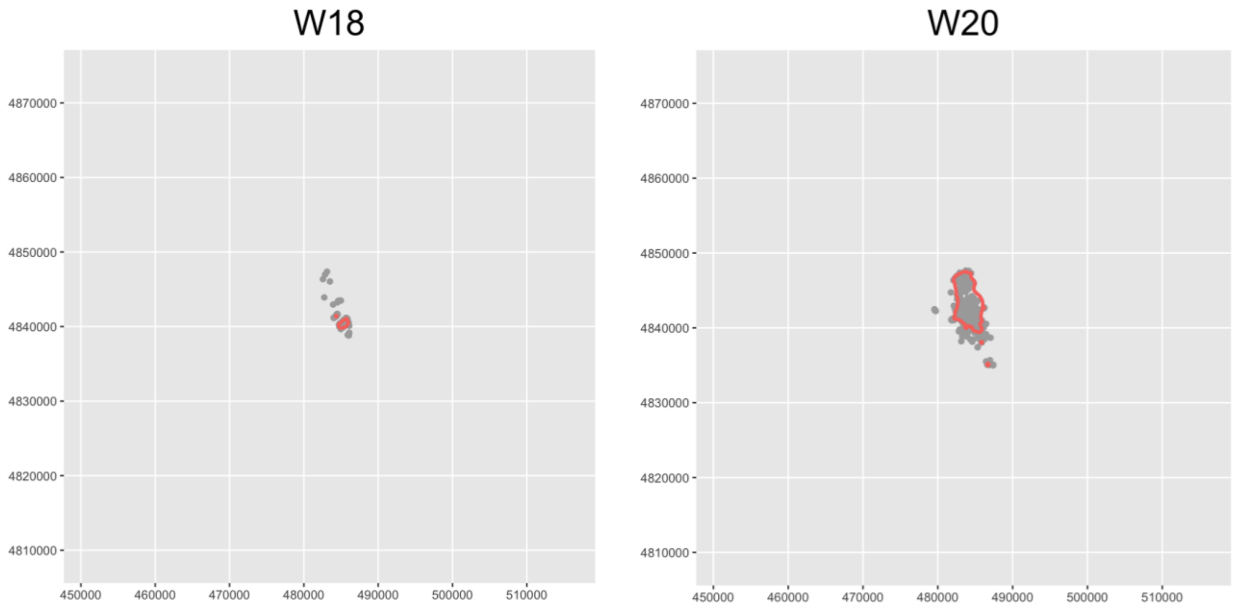

Figure S5. Estimated home ranges (95% utilization distributions) of reintroduced captive-rescued and wild Grey Crowned Cranes in Rwanda, calculated using dynamic Brownian Bridge Movement Models (dBBMM). Grey points represent individual GPS locations, and red contours indicate the 95% utilization distribution boundaries. Reintroduced cranes were released at Akagera NP, while wild cranes were tagged at Akanyaru Wetlands, Umusambi Village, Nyagatare, and Rugezi Marsh. All panels are uniformly scaled (70 km × 70 km) and centered on a consistent reference point within each release area. Axes are measured in meters, and each white grid line represents a distance of 10 km.
